# Phosphorylated and Phosphomimicking Variants May Differ—A Case Study of 14-3-3 Protein

**DOI:** 10.3389/fchem.2022.835733

**Published:** 2022-03-07

**Authors:** Aneta Kozeleková, Alexandra Náplavová, Tomáš Brom, Norbert Gašparik, Jan Šimek, Josef Houser, Jozef Hritz

**Affiliations:** ^1^ Central European Institute of Technology, Masaryk University, Brno, Czechia; ^2^ National Centre for Biomolecular Research, Faculty of Science, Masaryk University, Brno, Czechia; ^3^ Department of Chemistry, Faculty of Science, Masaryk University, Brno, Czechia

**Keywords:** 14-3-3, phosphorylation, phosphomimicking mutation, oligomeric state, dissociation constant

## Abstract

Protein phosphorylation is a critical mechanism that biology uses to govern cellular processes. To study the impact of phosphorylation on protein properties, a fully and specifically phosphorylated sample is required although not always achievable. Commonly, this issue is overcome by installing phosphomimicking mutations at the desired site of phosphorylation. 14-3-3 proteins are regulatory protein hubs that interact with hundreds of phosphorylated proteins and modulate their structure and activity. 14-3-3 protein function relies on its dimeric nature, which is controlled by Ser58 phosphorylation. However, incomplete Ser58 phosphorylation has obstructed the detailed study of its effect so far. In the present study, we describe the full and specific phosphorylation of 14-3-3ζ protein at Ser58 and we compare its characteristics with phosphomimicking mutants that have been used in the past (S58E/D). Our results show that in case of the 14-3-3 proteins, phosphomimicking mutations are not a sufficient replacement for phosphorylation. At physiological concentrations of 14-3-3ζ protein, the dimer-monomer equilibrium of phosphorylated protein is much more shifted towards monomers than that of the phosphomimicking mutants. The oligomeric state also influences protein properties such as thermodynamic stability and hydrophobicity. Moreover, phosphorylation changes the localization of 14-3-3ζ in HeLa and U251 human cancer cells. In summary, our study highlights that phosphomimicking mutations may not faithfully represent the effects of phosphorylation on the protein structure and function and that their use should be justified by comparing to the genuinely phosphorylated counterpart.

## Introduction

Protein phosphorylation is one of the most common post-translational modifications which has a unique role in regulation of protein function. Up to two thirds of the human proteome have been reported to be phosphorylated (Ardito et al., 2017) and approx. 3.5% of the human genome codes kinases and phosphatases, key players in the dynamic regulation of protein phosphorylation (Pearlman et al., 2011). Phosphorylation is responsible for the modulation of numerous cell processes including DNA transcription, apoptosis, metabolism, or antigen recognition (Braicu et al., 2019; Bernatik et al., 2020). Phosphorylation is also a focal point of many intricate signaling networks, cycles, feedback loops or cascades, as exemplified by the well-studied MAPK pathway or cell cycle checkpoints (Wei and Liu, 2002; Morrison, 2012).

The introduction of a phosphate group affects protein properties. The chemical properties of the protein surface are modulated by the large hydration shell and negative charge of the phosphate (Hunter, 2012; Laage et al., 2017). New hydrogen bonds may be formed, as well (Mandell et al., 2007). Phosphorylated residues are commonly present in binding motifs, effectively regulating entire protein-protein interactomes.

14-3-3 proteins are regulatory protein hubs expressed in all eukaryotes. The mammalian 14-3-3 protein family consists of seven isoforms (β, γ, ε, ζ, η, θ, and σ) that are involved in a variety of cellular processes, such as regulation of cell cycle, cellular growth and death, modulation of enzymatic activities and transcription factors, interaction with proteins of the cytoskeleton or signaling cascades (Mackintosh, 2004; Sluchanko and Gusev, 2010; Gardino and Yaffe, 2011; Obsilova and Obsil, 2020). 14-3-3 proteins form dimers, which is crucial for their function as protein scaffolds (Shen et al., 2003; Zhou et al., 2003; Messaritou et al., 2010). Interaction with more than 1,200 protein partners has been reported so far (Sluchanko and Bustos, 2019).

Phosphorylation plays a dual role in the 14-3-3 life cycle. First, phosphorylation of its partners significantly increases their binding affinity to a 14-3-3 dimer (Menzel et al., 2020; Munier et al., 2021). Second, the phosphorylation of 14-3-3 alters its own structure and function (Dubois et al., 1997; Tsuruta et al., 2004; Woodcock et al., 2003; Zhou et al., 2009). Phosphorylation of 14-3-3ζ at Ser58 has been proposed to impact the dimer-monomer equilibrium. However, either no effect on the oligomeric state (Powell et al., 2002), only partial dimer dissociation (Powell et al., 2003; Gu et al., 2006; Gerst et al., 2015) or complete monomerization (Woodcock et al., 2003; Kanno and Nishizaki, 2011; Civiero et al., 2017) were observed. Moreover, Ser58 phosphorylation and monomerization were shown to change the 14-3-3 protein properties, for example, monomeric 14-3-3 proteins proved higher chaperone-like activity than their dimeric counterparts (Sluchanko et al., 2012; Sluchanko et al., 2014). However, issues with preparation of the phosphorylated protein, e. g. incomplete phosphorylation or aggregation (Powell et al., 2003; Shen et al., 2003; Sluchanko and Uversky, 2015) obstructed the detailed description of changes in protein structure, properties, and interactions.

14-3-3ζ protein phosphorylated at Ser58 (hereafter pζ) was therefore often replaced by phosphomimicking and monomeric mutants (Sluchanko et al., 2008; Sluchanko et al., 2011b). To mimic the negative charge of the phosphate group, Ser58 was mutated to negatively charged amino acids, namely Asp (S58E) or Glu (S58D) (Powell et al., 2003; Sluchanko et al., 2008; Woodcock et al., 2018). Furthermore, the monomerization effect of Ser58 phosphorylation was substituted by mutations of conserved residues located at the dimeric interface. Several so-called monomeric mutants have been designed, namely septuple mutant E5K_L12Q_A13Q_E14R_Y82Q_K85N_E87Q (Tzivion et al., 1998; Shen et al., 2003), triple mutant L12Q_A13Q_E14R (Zhou et al., 2003; Messaritou et al., 2010; Sluchanko et al., 2011b) or our own double mutants L12E_M78K (hereafter ζm) and L12K_M78E (Jandova et al., 2018).

In general, phosphomimicking mutations have been reported to approximate the impact of phosphorylation with varying success (Thorsness and Koshland, 1987; Paleologou et al., 2008; Dephoure et al., 2013; Pérez-Mejías et al., 2020; Somale et al., 2020). To the best of our knowledge, in case of the 14-3-3 proteins, the properties of such mutants have never been compared to the phosphorylated protein, and thus their use has never been validated properly. Here we report a protocol for the preparation of 14-3-3ζ phosphorylated at Ser58 in high purity and sufficient amounts for biophysical analysis, and compare its characteristics, such as oligomeric state, thermal stability and hydrophobicity, with both phosphomimicking and monomeric mutants. Moreover, we present a novel double phosphomimicking mutant 14-3-3ζ S57D_S58D with its negative charge closer resembling the phosphate group.

## Materials and Methods

### Preparation of 14-3-3ζ Proteins

Six 14-3-3ζ protein constructs, namely 14-3-3ζ WT (abbreviated ζ), 14-3-3ζ phosphorylated at Ser58 (pζ), 14-3-3ζ L12E_M78K (ζm), 14-3-3ζ S58E (ζ_S58E), 14-3-3ζ S58D (ζ_S58D) and 14-3-3ζ S57D_S58D (ζ_S57D_S58D) were used in this study. All 14-3-3ζ protein constructs contained Cys-to-Ala mutations (i. e. C25A C189A) on the protein surface to prevent the formation of intermolecular disulphide bridges in solution (Hritz et al., 2014). cDNA of the 14-3-3ζ proteins, containing an N-terminal 6×His-tag separated by a Tobacco Etch Virus (TEV) protease cleavage site, was expressed from a pET15b plasmid (Novagene). The monomeric mutant ζm and the double mutation L12E_M78K was designed previously (Jandova et al., 2018). The mutations S58E, S58D and double mutation S57D_S58D were incorporated using the PCR mutagenesis protocol of Liu and Naismith (2008). For fluorescence experiments, ζ_S58E_Ntail construct was prepared by adding the SVDACKGSSGG sequence at the ζ_S58E N-terminus. cDNA of all 14-3-3ζ mutants was verified by DNA sequencing (SeqMe, Czech Republic). Except for pζ, all 14-3-3ζ constructs were expressed in the bacterial *E. coli* strain BL21(DE3) CodonPlus RIL (Stratagene) and purified according to a previously optimized protocol (Hritz et al., 2014).

### Preparation of 14-3-3ζ Phosphorylated at Ser58

14-3-3ζ phosphorylated at Ser58 was prepared by co-expression of ζ with the catalytic subunit of mouse protein kinase A (hereafter shortly PKA) in *E. coli*. A pACYCduet-1 plasmid, bearing the 6×His-PKA cDNA and chloramphenicol resistance (kindly provided by prof. Nikolai Sluchanko, Russian Academy of Sciences, Moscow), was transformed into *E. coli* BL21(DE3) (Novagen) using heat shock at 42°C for 30 s. The transformed cells were then made competent using the Inoue method (Inoue et al., 1990) and used for subsequent transformation of the pET15b-14-3-3ζ plasmid (ampicillin resistance).

100 mL Lysogeny broth (LB) overnight culture was pelleted, resuspended in fresh medium, and transferred into 2 L of sterile LB supplemented with ampicillin and chloramphenicol. The culture was incubated at 37°C and 180 RPM until OD_600_ approached ∼0.9, when protein expression was induced by 0.5 mM IPTG and expression continued for 4 h under the same conditions. Cells were harvested by centrifugation at 5,000 g, 4°C for 15 min, the bacterial pellet was resuspended in 20 mL of 50 mM Tris-HCl, 150 mM NaCl, 20 mM imidazole, 10% glycerol, pH 8 and stored at −80°C.

Rapidly thawed cell pellet was supplemented with 0.3 mg/mL lysozyme and sonicated for 30 min on ice (50 W, 20 kHz, 1 s pulse duration, 5 s delay) (QSonica Q700 Sonicator). Cell debris was pelleted by centrifugation at 21,000 g, 4°C for 60 min and the cleared supernatant was loaded onto a Ni^2+^ immobilized metal affinity chromatography (IMAC) column (5 mL HisTrap HP, GE Healthcare) equilibrated in 50 mM Tris-HCl, 500 mM NaCl, 3 mM NaN_3_, pH 8. His-tagged proteins were eluted with a linear gradient of 20–500 mM imidazole in one column volume (CV). Eluted sample was diluted 2-times with 20 mM Tris-HCl pH 8 to reduce imidazole and salt concentration and 6×-His-TEV protease (in-house prepared recombinant protein) was added in a molar ratio 1 : 20 (TEV: 14-3-3ζ monomer). The mixture was dialyzed against TBS (50 mM Tris-HCl, 150 mM NaCl, 3 mM NaN_3_, pH 8) at 4°C overnight. The next day, His-tagged enzymes (TEV and PKA) and cleaved His-tag were removed from 14-3-3ζ by another Ni^2+^ IMAC step. 14-3-3ζ protein was collected in the flow-through and diluted 2-times with 20 mM Tris-HCl pH 8 to decrease the ionic strength of the solution. Subsequently, the protein sample was applied to an anion-exchange chromatography (AEX) column (5 mL HiTrap Q HP, GE Healthcare) equilibrated in 20 mM Tris-HCl, 3 mM NaN_3_, pH 8. 14-3-3ζ proteins (ζ and pζ) were separated during elution with a slow linear gradient of NaCl (200–500 mM) in six CVs. The pζ fraction, which eluted at conductivity ∼25 mS/cm, was collected and concentrated to 4 mL. Finally, the pζ sample was applied onto a TBS-equilibrated size-exclusion chromatography (SEC) column (HiLoad 16/600 Superdex75 pg, GE Healthcare) to remove any remaining impurities.

Protein purity was evaluated by SDS-PAGE. Accurate protein molecular weight (M_W_) was verified by MALDI-TOF mass spectrometry (MS) (ultrafleXtreme, Bruker). The precise position of the phosphorylation site was identified using trypsin proteolysis followed by LC-MS/MS (RSLC nano, Dionex; Orbitrap Fusion Lumos, Thermo Scientific). Protein concentration was determined spectrophotometrically at 280 nm using NanoDrop 2000/2000c Spectrophotometer (ThermoFisher). 14-3-3ζ protein extinction coefficient (27,390 L mol^−1^ cm^−1^) was determined using the ProtParam tool (Gasteiger et al., 2005).

### Circular Dichroism Spectroscopy (CD)

Far-UV CD measurements were performed on a J-815 spectrometer (Jasco) at 20°C in 1-mm Quartz cuvette (Hellma Analytics). CD spectra of 0.2 mg/mL (7.3 μM) 14-3-3ζ proteins in 20 mM sodium phosphate buffer pH 7.4 were acquired as 5 accumulations in the wavelength range 185–260 nm with 1 nm step at scanning speed 50 nm/min. Subsequently, buffer signal was subtracted, and data were converted from circular dichroism units to mean residue molar ellipticity (MRE) to account for precise protein concentration. The presence of secondary structural elements was evaluated using DichroWeb (K2D programme) (Andrade et al., 1993) and K2D3 programme (Louis-Jeune et al., 2012).

### Native-PAGE

25 μM 14-3-3ζ protein samples in 50 mM Tris-HCl, 300 mM NaCl, 3 mM NaN_3_, pH 8 were equilibrated at 37°C for 20 min. Afterwards, native-PAGE on a 12.5% gel was performed at 90 V for 4.5 h on ice.

### Analytical Ultracentrifugation (AUC)

Sedimentation velocity (SV) experiment was performed with 1 mg/mL (36.5 μM) 14-3-3ζ proteins in 50 mM Tris-HCl, 300 mM NaCl, 3 mM NaN_3_, pH 8. Measurements were conducted using ProteomeLab XL-I ultracentrifuge (Beckman Coulter) in a 4-hole An-60 Ti rotor at 20°C and 50,000 RPM with continuous absorbance detection at 286 nm. 200 scans in 5-min intervals were acquired with 0.003 cm radial size increment. Partial-specific volume of 14-3-3ζ, solvent density and viscosity were predicted using Sednterp^1^ software. The SV data from 100 to 150 scans corresponding to complete sample sedimentation were fitted using Sedfit v15.01c (Schuck, 2000) with a continuous c(s) distribution model. M_W_ of particles was estimated based on the Svedberg equation. c(s) distributions were normalized and plotted using the GUSSI programme version 1.4.2 (Brautigam, 2015).

### Analytical size-exclusion chromatography with right-angle light scattering detection (SEC-RALS).

*For comparison of the oligomeric states:* Prior to measurement, 1 mg/mL (36.5 μM) 14-3-3ζ proteins in PBS (10 mM Na_2_HPO_4_, 1.8 mM KH_2_PO_4_, 137 mM NaCl, 2.7 mM KCl, 1 mM NaN_3_, pH 7.4) were centrifuged at 14,000 g, 4°C for 10 min. SEC-RALS measurements were performed on an OmniSEC instrument (Malvern Panalytical). 50 μL of each protein was injected onto a 13-mL Zenix SEC 300 gel filtration column (Sepax Technologies) equilibrated in PBS and analysis was performed in triplicates at 20°C and flow rate 0.7 mL/min. UV detection at 254 nm was used to monitor the separation. Data were evaluated using OMNISEC software 11.21 (Malvern Panalytical). M_W_ of particles was calculated from the intensity of the scattered light, measured at right angle for maximal sensitivity, based on the Rayleigh equation.

*For estimation of the dimerization dissociation constant (K*_*D*_) *of pζ, ζm, ζ_S58D and ζ_S57D_S58D:* 5 mg/mL (183 μM) pζ and ζm or 4.5 mg/mL (164 μM) ζ_S58D and ζ_S57D_S58D were analyzed using the same setup as above (in triplicates).

### Förster Resonance Energy Transfer (FRET) Assay

ζ_S58E_Ntail was fluorescently labelled at position Cys5 with AlexaFluor647-C5-maleimide (AF647) and AlexaFluor488-C2-maleimide (AF488) (Thermo Fisher Scientific), as described in Trošanová et al. (2022). Fluorescence measurements were performed on a FluoroLog-3 Modular Spectrofluorometer (HORIBA Jobin Yvon) in 10.00-mm Quartz glass cuvette (High Precision Cell, Hellma Analytics) with a magnetic stirrer. Prior to measurement, the cuvette was treated with 15 mg/mL BSA for 30 min to prevent adhesion of fluorescently labelled ζ_S58E_Ntail to the cuvette walls. Measurements were conducted in 20 mM sodium phosphate pH 6.8 at 15°C with λ_ex_ = 470 nm (slits 2 mm) and λ_em_ = 666 nm (slits 5 mm). Data were collected each 30 s with 0.5 s integration time.

FRET assay was performed as detailed in Trošanová et al. (2022). In brief, first, fluorescence signal of 200 nM ζ_S58E_Ntail labelled with AF647 was recorded for ∼20 min. Afterwards, 200 nM ζ_S58E_Ntail labelled with AF488 was added and FRET was initiated. After stabilization of the fluorescence signal (∼60 min), non-labelled ζ_S58E in 100-fold excess was added to disrupt the FRET dimers. Acquired FRET kinetic profiles were fitted, as described in Trošanová et al. (2022) and dissociation constant K_D_ and dissociation rate constant k_off_ were extracted.

### Differential Scanning Calorimetry (DSC)

DSC thermograms of 1 mg/mL (36.5 μM) 14-3-3ζ proteins dialyzed into PBS were acquired using Microcal PEAQ-DSC Automated calorimeter (Malvern Panalytical). Prior to measurement, samples were centrifuged at 18,000 g and 4°C for 10 min. Reference cell was filled with a corresponding buffer after dialysis. DSC measurements were performed in triplicates, and each was composed of three periods: heating (20–90°C, heating rate 1°C/min), cooling (90–20°C, −1°C/min) and final heating (20–90°C, 1°C/min). Buffer measurements were performed prior to each triplicate and these data were then subtracted from the protein scan to eliminate the buffer signal. Second and third heating scans were used for assessment of the denaturation reversibility.

Thermodynamic parameters of 14-3-3ζ proteins were evaluated using MicroCal PEAQ-DSC software (Malvern Panalytical). Thermogram of the buffer was subtracted from the thermogram of the protein and the ‘progress’ baseline was defined. Data were fitted with a non-two-state model used for determination of enthalpies of denaturation and melting temperatures.

### Differential Scanning Fluorimetry (nanoDSF)

Thermal stability of 1 mg/mL (36.5 μM) 14-3-3ζ proteins was measured in PBS or in 20 mM HEPES pH 8.0 with 0/50/200/400/600 mM NaCl/Na_2_SO_4_/Na_2_HPO_4_ to study the effect of different anions. Measurements were performed in triplicates using a Prometheus NT.48 instrument (NanoTemper Technologies) in the temperature range 20–80°C with a temperature slope of 1°C/min and at excitation power 75%. Protein unfolding was monitored by fluorescence intensity measured at 330 and 350 nm. Subsequently, melting temperature (T_m_) was determined from the first derivative of fluorescence ratio (330/350) (Alexander et al., 2014).

### BisANS Binding Assay

BisANS (4,4′-Dianilino-1,1′-binaphthyl-5,5′-disulfonic acid dipotassium salt) powder (Sigma-Aldrich) was dissolved in MilliQ water and its concentration was determined spectrophotometrically using NanoDrop 2000/2000c Spectrophotometer (ThermoFisher) based on BisANS absorbance at 385 nm and known extinction coefficient (ε_385nm,water_ = 16,790 L mol^−1^ cm^−1^) (Sharma et al., 1998). Fluorescence measurements were performed at 37°C on a FluoroLog-3 Modular Spectrofluorometer (HORIBA Jobin Yvon) in a 10.00-mm Quartz glass cuvette (High Precision Cell, Hellma Analytics) with a magnetic stirrer. 1 μM 14-3-3ζ proteins in 1 mL of PBS were titrated with BisANS to final concentration 1–30 μM. After each addition of BisANS, the system was equilibrated for 6 min and then fluorescence of BisANS was excited at 385 nm and detected in the range 400–700 nm with entrance/intermediate/exit slit widths set to 1.5 mm. BisANS fluorescence intensity at 495 nm was used for assessment of protein hydrophobicity. Between measurements of different proteins, the cuvette was cleaned with 3 M HNO_3_ for 30 min while stirring to remove all potential contaminants.

### Cell Culture Maintenance

HeLa 1.3 cells and U251 cells were maintained at 37°C with 5% CO_2_ in Dulbecco’s Modified Eagle Medium (Gibco) supplemented with 10% Fetal Bovine Serum (Capricorn), Non-essential amino acids (Gibco), Penicillin-Streptomycin (Sigma-Aldrich), and L-glutamine (Gibco). For the experiments, cells were grown on #1.5 round coverslips in a 24-well plate with a seeding density of 5 × 10^4^ cells per well.

### Immunofluorescence Protocol

The day after seeding, cells were washed three times with PBS and fixed with 4% formaldehyde (Serva) in PBS for 10 min at room temperature followed by a wash with PBS and permeabilization in PBS containing 0.1% Triton X-100 for 10 min at room temperature. Cells were then washed with PBS and blocked in blocking buffer (5% goat serum (Biosera) in PBS) for 1 h at room temperature. After blocking, cells were incubated with a primary antibody (Anti-14-3-3ζ (phospho-S58) antibody (ab51109, Abcam) and 14-3-3ζ Antibody (MA5-37641, Invitrogen)) diluted in blocking buffer at 1:200 for 1 h at room temperature. Then the cells were washed 4 × 5 min with PBS supplemented with 0.05% Tween 20 (PBS-T) and incubated with secondary anti-mouse antibody conjugated with Alexa Fluor 488 (Goat Anti-Mouse IgG H&L (Alexa Fluor^®^ 488), ab150113) and anti-rabbit antibody conjugated with Alexa Fluor 594 (Goat Anti-Rabbit IgG H&L (Alexa Fluor^®^ 594), ab150080) diluted in blocking buffer at 1:250 for 1 h at room temperature. Finally, the cells were washed 4 × 5 min with PBS-T and mounted on microscope slides using ProLong Omega Glass (Invitrogen) with DAPI staining for nuclear DNA.

### Fluorescence Microscopy

Experiments were performed with a wide field epi-fluorescence microscope Zeiss AxioImager Z2 using appropriate filters and dedicated software ZEN studio. The oil-immersion Plan-APO objective 63× was used to capture the desired image. The images were deconvoluted using the ZEN studio imaging module and analyzed using ImageJ.

## Results

### Preparation of 14-3-3ζ Phosphorylated at Ser58

**FIGURE 1:**
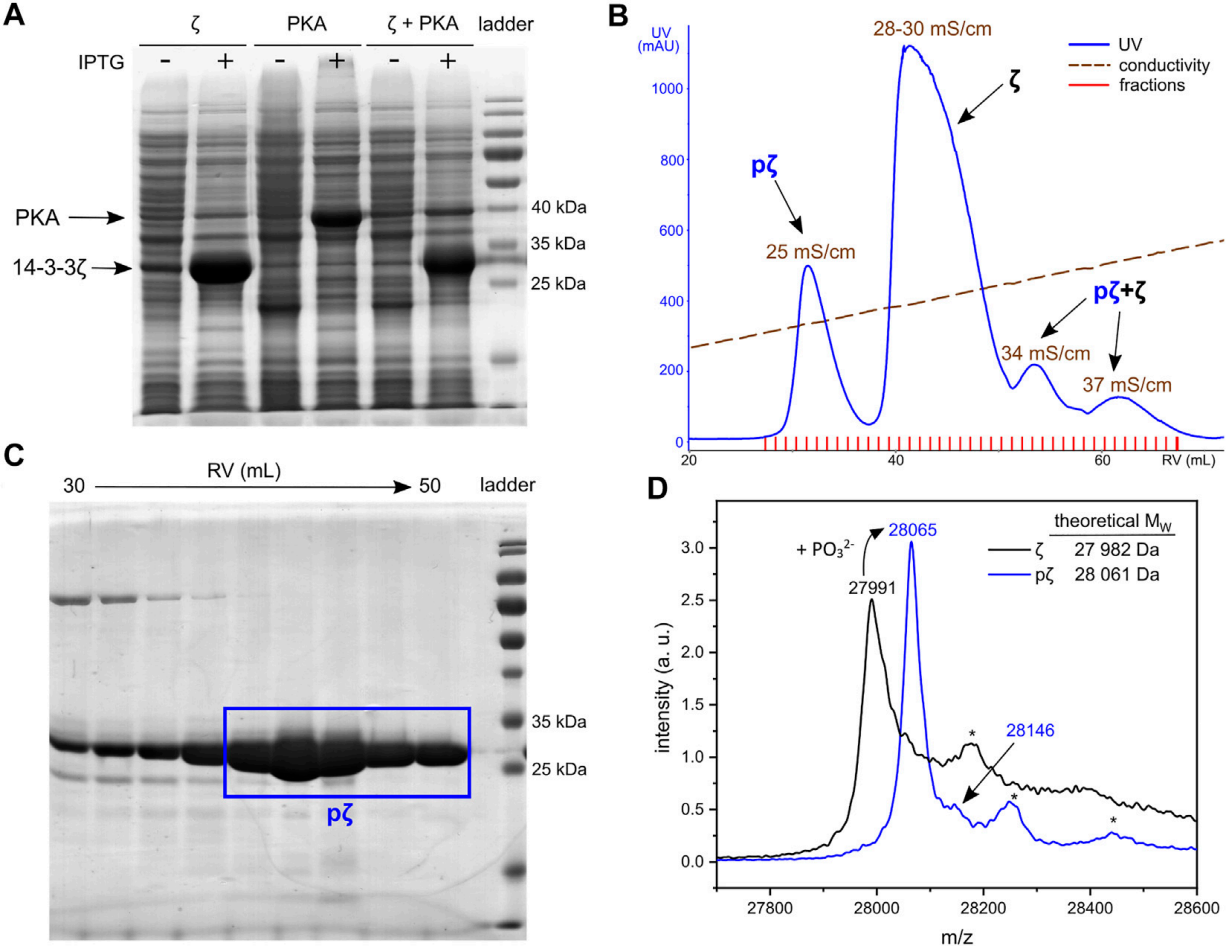
Expression and purification of pζ. **(A)** SDS-PAGE analysis of 14-3-3ζ and PKA co-expression. Cultures without (−) and with (+) induction with IPTG are shown. **(B)** Chromatogram of AEX elution. Average conductivity characterizing the eluted peaks is highlighted. **(C)** SDS-PAGE analysis of the SEC purification step. Fractions corresponding to the final pζ sample are depicted in a blue rectangle. **(D)** MALDI-TOF MS spectrum of purified ζ (in black) and pζ (in blue). Asterisk denotes a protein adduct with the MALDI matrix (ferulic acid, +176).

14-3-3ζ phosphorylated at Ser58 was obtained by co-expression of 14-3-3ζ and protein kinase A (PKA) in *E. coli*. The catalytic subunit of mouse PKA was employed due to its high specificity for the PKA recognition motif ‘RRX**S**Y’ (Y stands for hydrophobic residue), which corresponds to the sequence surrounding Ser58 (i. e. RRS**S** ^**58**^W) (Ma et al., 2005; Woodcock et al., 2010; Sluchanko and Uversky, 2015). The bacterial *E. coli* strain BL21(DE3) was transformed consecutively with the cDNA of PKA and 14-3-3ζ, located on compatible plasmids. 14-3-3ζ and PKA were then co-expressed (Figure 1A) and Ser58 was phosphorylated *in vivo*. Despite extensive screening of suitable conditions (e. g. temperature, length of expression, culture medium composition), the level of Ser58 phosphorylation reached only approx. 10–20%, and the two forms of protein (ζ, pζ) needed to be separated during the purification process. The optimized protocol is detailed in Methods.

Phosphorylated 14-3-3ζ was separated from the non-phosphorylated ζ by fine-tuning the anion-exchange chromatography (AEX). A linear salt gradient (200–500 mM NaCl in 6 CVs) enabled good separation of pζ from ζ (Figure 1B). The pζ fraction was eluted at conductivity ∼25 mS/cm, non-phosphorylated ζ at 28–30 mS/cm. Minor fractions which eluted at conductivity 34 and 37 mS/cm contained a mixture of both pζ and ζ and were discarded (Supplementary Figure S1). Remaining impurities were removed from the pζ fraction by size-exclusion chromatography (SEC) (Supplementary Figure S2). Finally, we obtained ∼7 mg of pure pζ from 1 L of LB medium.

Protein purity and identity was verified by SDS-PAGE and mass spectrometry. Based on SDS-PAGE (Figure 1C), the selected SEC fractions contain protein of high purity that migrates at M_W_ around 25–30 kDa, which corresponds to calculated M_W_ of pζ (28,061 Da). Accurate protein M_W_ was confirmed by MALDI-TOF MS (Figure 1D). An additional peak with a higher m/z (+79 for the phosphate group) was also observed and indicated a minor fraction of doubly phosphorylated protein. Trypsin digestion and LC-MS/MS showed that pζ was fully phosphorylated at Ser58 and the residual phosphorylation was located at Ser28 (∼4%) (for details see Supplementary Material).

### Selected 14-3-3ζ Mutants for Comparison With pζ

**FIGURE 2:**
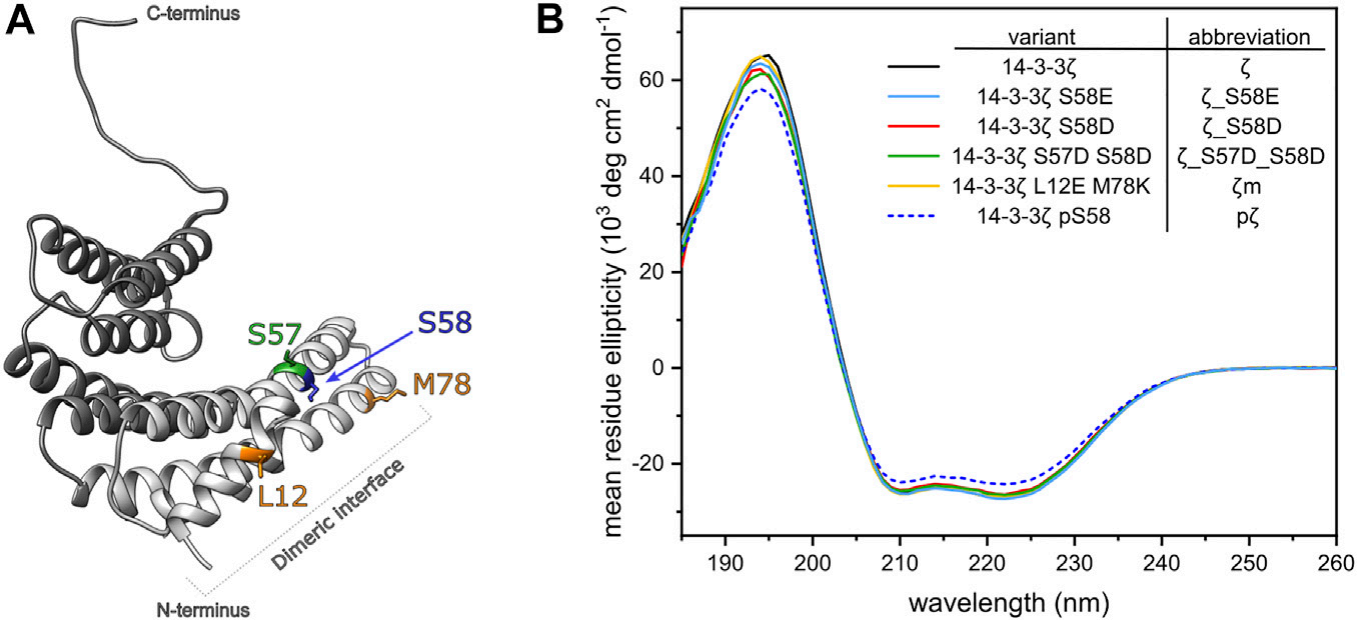
Design and secondary structure analysis of 14-3-3ζ phosphomimicking and monomeric mutants. **(A)** Structural model of the 14-3-3ζ monomeric subunit with the modified residues highlighted in color. Helices forming the dimeric interface are depicted in light grey. Atom coordinates were adopted from Kostelecky et al. (2009) and Nagy et al. (2017). **(B)** Far-UV CD spectrum of 14-3-3ζ variants. For clarity, 14-3-3ζ variants, used in this study, and their abbreviations are listed in the legend.

Phosphomimicking mutants of 14-3-3 proteins have been used extensively to study the impact of Ser58 phosphorylation on protein structure and interactions (Powell et al., 2002, 2003; Gu et al., 2006; Sluchanko et al., 2008; Sluchanko et al., 2011a; Woodcock et al., 2018). In this study, we prepared the previously reported phosphomimicking mutants of 14-3-3ζ protein, namely S58D (ζ_S58D) and S58E (ζ_S58E). Moreover, we wanted to address the problem that Asp and Glu possess a lower negative charge than the phosphate group. At physiological pH ∼7.4, the negative charge of phospho-residues is dominantly –2e [pK_a2_ (pSer) = 5.6; pK_a2_ (pThr) = 5.9], whereas Asp and Glu are only singly charged (Xie et al., 2005; Pearlman et al., 2011; Hunter, 2012). Inspired by Strickfaden et al. (2007), we designed a novel double mutant ζ_S57D_S58D with two phosphomimicking mutations at neighboring residues, to mimic the phosphate’s negative charge more realistically. Since phosphorylation at Ser58 and the corresponding phosphomimicking mutations have been proposed to induce monomerization (Powell et al., 2003; Woodcock et al., 2003; Sluchanko et al., 2008), we employed our monomeric mutant ζm (Jandova et al., 2018), as a monomer control. Positions of modified residues at the dimeric interface of the constructs used in this study are shown in Figure 2A and abbreviations of 14-3-3ζ variants are listed in Figure 2B.

To analyze whether the mutations or Ser58 phosphorylation result in any change of 14-3-3ζ secondary structure, we employed far-UV CD spectroscopy. CD spectra (Figure 2B) showed that all studied ζ variants retained the α-helical structure characteristic to 14-3-3 proteins. Abundance of the individual secondary structural elements was determined by deconvolution of the acquired CD curves using two programmes, namely K2D (Andrade et al., 1993) and K2D3 (Louis-Jeune et al., 2012). The portion of α-helices was estimated to be 79% and 85%, according to K2D and K2D3, respectively. Deviations in α-helical content between proteins were lower than ±1%. pζ protein displayed the same CD profile, but the MRE magnitude was decreased by approx. 10%, compared to the other 14-3-3ζ variants.

### Phosphorylation and Phosphomimicking Mutations Have Different Impact on 14-3-3ζ Oligomeric State

The phosphorylation of Ser58 has been proposed to induce monomerization of 14-3-3ζ protein (Woodcock et al., 2003). Therefore, we inspected the impact of phosphorylation and phosphomimicking mutations on the dimer-monomer equilibrium in detail. We aimed to compare the dimer-monomer distribution of 14-3-3ζ variants at biologically relevant concentrations. We calculated the concentration of the 14-3-3ζ isoform in the human brain based on the equation proposed by Gogl et al. (2021) and data from the PAXdb database^2^. We obtained the value of 25 μM and, as a result, we examined the 14-3-3ζ oligomeric states at 25 and 36.5 μM (equals to the standard 1 mg/mL) concentrations.

**FIGURE 3:**
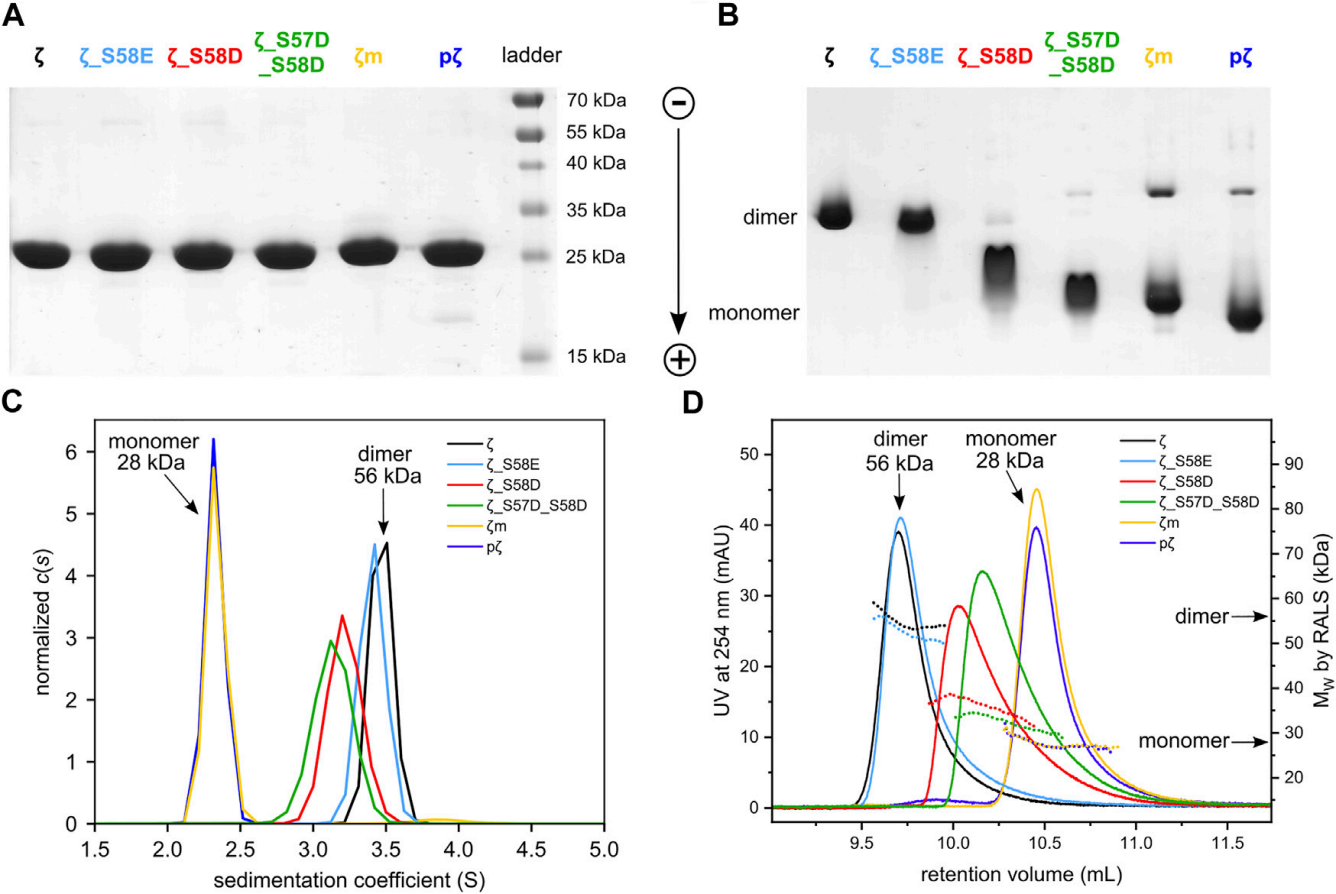
Differences in oligomeric state between 14-3-3ζ variants. **(A)** SDS-PAGE analysis of 25 μM 14-3-3ζ variants comparing their electrophoretic mobility under denaturing conditions, **(B)** native-PAGE of 25 μM 14-3-3ζ proteins, **(C)** AUC of 36.5 μM 14-3-3ζ proteins, **(D)** SEC-RALS of 36.5 μM 14-3-3ζ proteins.

First, to verify the purity and identity of tested 14-3-3ζ variants, we employed SDS-PAGE and mass spectrometry. As expected, all 14-3-3ζ variants exhibit similar electrophoretic mobility in SDS-PAGE. The migration rates correspond to the approx. M_W_ of 28 kDa, which is in agreement with the calculated M_W_ of a 14-3-3ζ monomer (Figure 3A). Comparable M_W_ values were also confirmed by MALDI-TOF MS (Figure 1D, Supplementary Figure S3).

Next, the oligomeric states of 14-3-3ζ variants were analyzed using native-PAGE, AUC and SEC-RALS (Figures 3B–D; Table 1). In all cases, the phosphomimicking mutants exhibited distinct behavior from pζ. The electrophoretic mobility, sedimentation coefficient (3.6 S) as well as retention volume (9.7 mL) of ζ_S58E was comparable to non-phosphorylated ζ (3.7 S, 9.7 mL) suggesting that ζ_S58E occurs predominantly in the dimeric state at physiological concentration. However, a lower M_W_ (51 kDa) determined from SEC-RALS data and sedimentation coefficient (Table 1) implied that the S58E mutation partially destabilizes the dimer with respect to the ζ variant. The phosphomimicking mutants ζ_S58D and ζ_S57D_S58D formed diffuse bands of intermediate mobility in native-PAGE (Figure 3B), indicating a more substantial shift of the dimer-monomer equilibrium towards monomers. Their sedimentation coefficient (∼3.4 S), elution position (10.0–10.2 mL) and determined M_W_ (32–44 kDa) corresponded neither to a dimer (3.7 S, 9.7 mL, 56 kDa) nor a monomer (2.5 S, 10.5 mL, 28 kDa) at these concentrations (Table 1). At the same time, the double mutant ζ_S57D_S58D appeared to be more monomeric than ζ_S58D.

**TABLE 1:** AUC and SEC-RALS parameters characterizing the oligomeric state of 14-3-3ζ proteins. The oligomeric state of proteins (36.5 μM) was analyzed at 20°C.

14-3-3	AUC		SEC-RALS	
	s_20,w_ (S)	Apparent M_W_ (kDa)	RV (mL)	Apparent M_W_ (kDa)
ζ	3.71	56.4	9.70	54.5
ζ_S58E	3.64	56.3	9.72	50.7
ζ_S58D	3.44	43.7	10.03	34.9
ζ_S57D_S58D	3.35	38.6	10.16	32.0
ζm	2.50	27.0	10.47	27.7
pζ	2.47	28.1	10.46	27.2

On the contrary, phosphorylation was observed to shift the dimer-monomer equilibrium towards monomers more significantly. pζ characteristics were similar to the monomeric mutant ζm (2.5 S, 10.5 mL, 28 kDa). Moreover, pζ migrated faster in native-PAGE than ζm (Figure 3B), presumably due to additional negative charge (−2e per monomeric unit) introduced by the phosphate group.

Since the populations of dimeric and monomeric states are dependent on the actual protein concentration, we next aimed to describe the dimer-monomer equilibrium of individual 14-3-3ζ variants quantitatively, using the dimerization dissociation constants (Table 2). The dimer-monomer equilibrium can be described as

M+M⇄D

where M corresponds to monomer and D to dimer. The dimerization dissociation constant (K_D_) can be then expressed as:

$$\begin{array}{c} K_{D}=\frac{\left[M\right]\left[M\right]}{\left[D\right]} \end{array}$$ (1)

where [M] and [D] stand for molar concentrations of monomer and dimer, respectively.

**TABLE 2:** Dissociation constants of dimerization determined for 14-3-3ζ variants. Populations of monomers and dimers at 25 μM concentrations are included.

14-3-3	K_D_	(M) (%)	(D) (%)
ζ_S58E	(0.35 ± 0.25) μM	8.02	91.98
ζ_S58D	(132 ± 7) μM	77.34	22.66
ζ_S57D_S58D	(348 ± 19) μM	88.70	11.30
ζm	(4.6 ± 0.1) mM	98.94	1.06
pζ	(7.6 ± 0.8) mM	99.35	0.65

**FIGURE 4:**
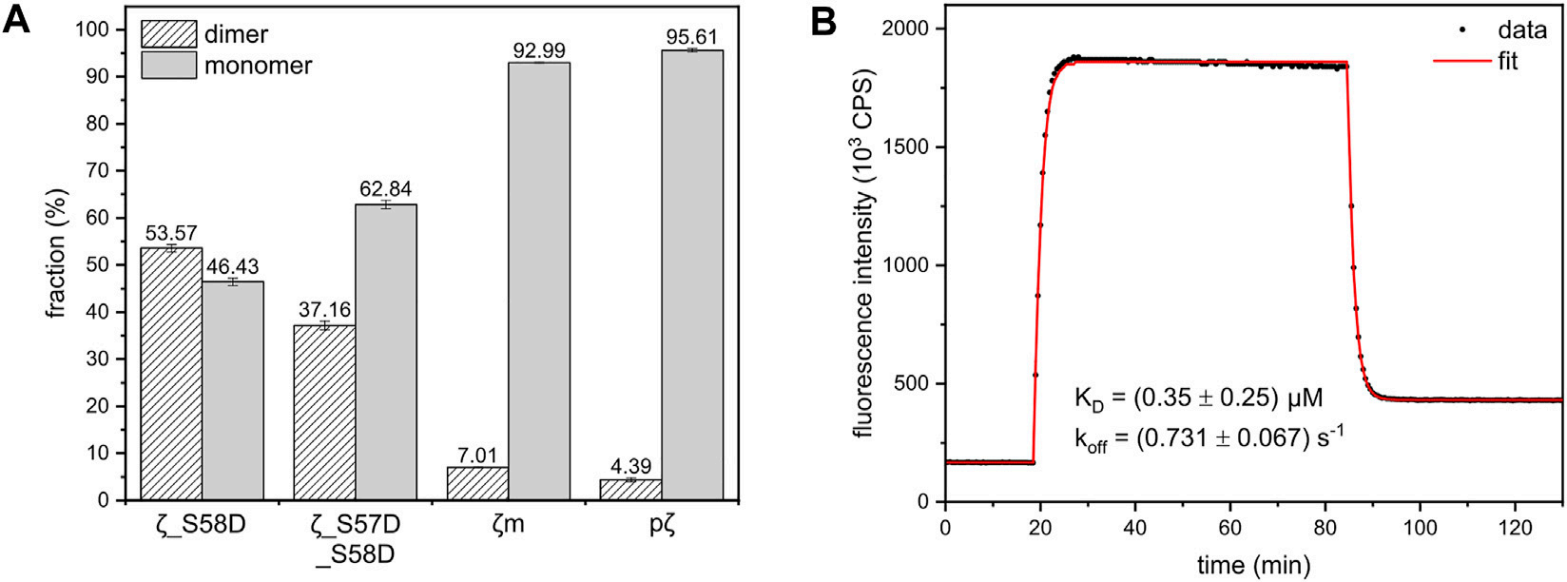
Dimer-monomer distributions and FRET experiment used for calculation of the dimerization K_D_ of 14-3-3ζ variants. **(A)** The populations of 14-3-3ζ proteins (*x*-axis) in the dimeric and monomeric state determined from SEC-RALS experiments (Supplementary Figure S4) as described in the text. The dimer-monomer populations for 164 μM ζ_S58D and ζ_S57D_S58D mutants and 183 μM ζm and pζ variants at 20°C are shown. **(B)** FRET kinetic profile of ζ_S58E dimerization at 15°C. The experimental data (black dots) were fitted (red line), as in Trošanová et al. (2022), to obtain K_D_ and k_off_ values.

To determine the dimerization constants of the 14-3-3ζ variants, we employed SEC-RALS and fluorescence assay based on FRET. Since ζm and pζ were observed to adopt both dimeric and monomeric states in native-PAGE and SEC-RALS (Figure 3B and Figure 3D), the dissociation constants of ζm and pζ were estimated directly from the distribution of proteins in the monomeric and dimeric state. The protein concentration in the dimeric [D] and monomeric [M] peak in SEC-RALS (Supplementary Figure S4) was determined based on known total protein concentration and protein percentage in individual peaks (Figure 4A).

The dissociation constants of ζ_S58D and ζ_S57D_S58D were calculated from M_W_ determined by SEC-RALS (Supplementary Figure S4). From the measurement at 36.5 μM concentration (Figure 3D), we saw that these phosphomimicking mutants exist as a heterogenous mixture of dimer and monomer. Since particles of different size contribute to RALS proportionally to their population, the observed M_W_ is a weighted average of molecular weights corresponding to dimer and monomer. Considering the following Equation 2, we calculated the fractions of proteins in the dimeric (D) and monomeric state (M) (Figure 4A).

$$\begin{array}{c} M_{W,obs}=M_{W,\zeta }\left(D\right)+M_{W,p\zeta }\left(M\right) \end{array}$$ (2)

where M_W,obs_ corresponds to M_W_ of ζ_S58D and ζ_S57D_S58D determined from RALS, M_W,ζ_ and M_W,pζ_ stand for M_W_ of ζ (dimer) and pζ (monomer) determined from RALS, respectively. Afterwards, knowing the total protein concentration, we determined [D] and [M] and finally the K_D_.

In contrast to other 14-3-3ζ variants, the dissociation constant of ζ_S58E could not be obtained from SEC-RALS due to high preference of ζ_S58E for the dimeric form at selected concentrations (1–5 mg/mL). A more sensitive approach using much lower protein concentrations was required. Therefore, we applied a kinetic assay based on FRET. First, ζ_S58E was specifically labelled with the fluorescent donor as well as the acceptor. Afterwards, time dependence of FRET intensity originating from the formation of ζ_S58E dimers was measured and fitted (Figure 4B) by a set of differential equations, as described in Trošanová et al. (2022). The best fit values for the dimerization K_D_ and dissociation rate constant (k_off_) altogether with their errors were extracted from heatmap analysis (Supplementary Figure S5).

From the definition equation of K_D_ (Eq. 1) and mass conservation equation:

$$\begin{array}{c} \left[M\right]+2\left[D\right]=c_{tot} \end{array}$$ (3)

where c_tot_ corresponds to total molar concentration of proteins (per 14-3-3ζ monomer), we can calculate the [M] at any protein concentration as follows:

$$\begin{array}{c} \left[M\right]=\frac{-K_{D}+\sqrt{K_{D}^{2}+8K_{D}c_{tot}}}{4} \end{array}$$ (4)

In Table 2, for illustration, we provide calculated populations of dimers and monomers at physiological concentration of 14-3-3ζ (25 μM).

### Phosphorylation and Monomerization Decreases Thermal Stability

**FIGURE 5:**
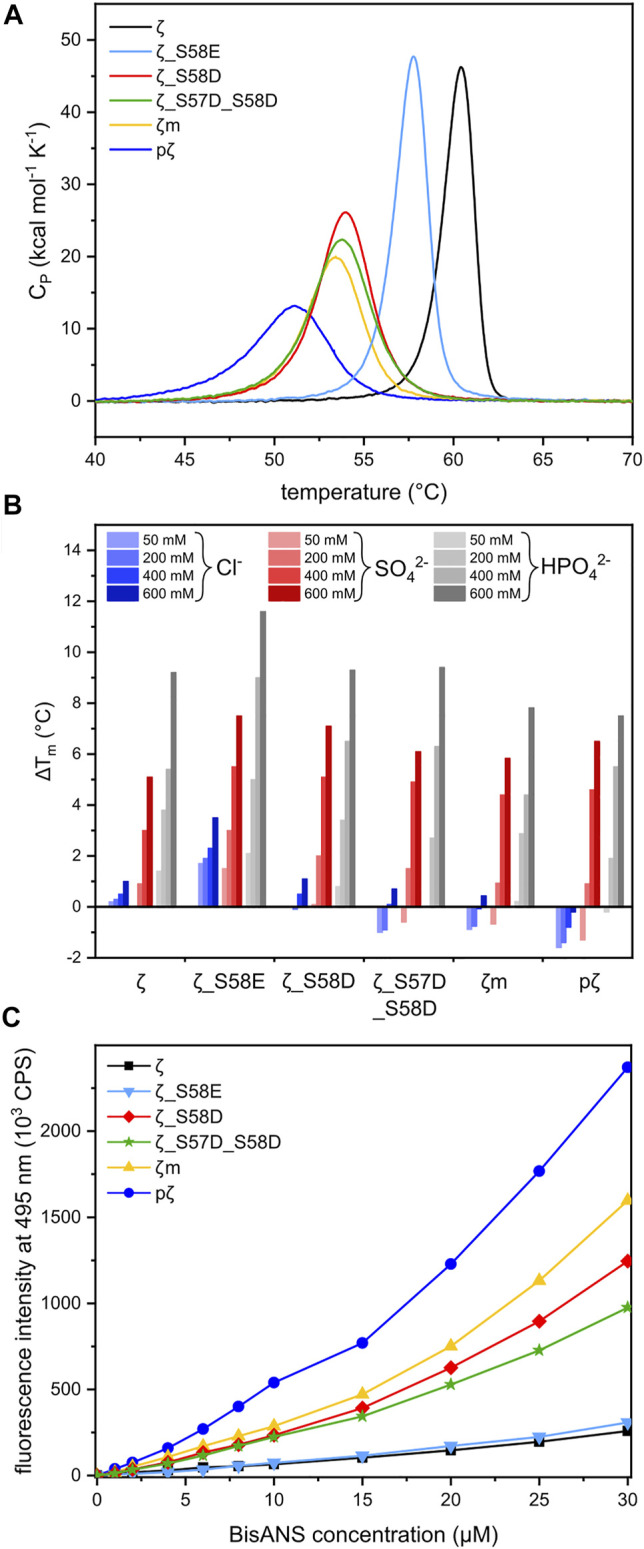
Thermal stability and hydrophobicity of 14-3-3ζ variants. **(A)** DSC thermograms of 14-3-3ζ proteins demonstrating differences in T_m_ and global unfolding. T_m_ values were determined from the peak maxima. **(B)** Diverse effects of selected sodium salts on the stability of 14-3-3ζ variants. T_m_ of each protein variant in 20 mM HEPES pH 8 (Supplementary Table S1) was set as a reference (0°C). **(C)** BisANS fluorescence intensity at 495 nm used for comparison of 14-3-3ζ hydrophobicity.

To assess the impact of phosphorylation and phosphomimicking mutations on 14-3-3ζ protein thermal stability, we employed nanoDSF and DSC measurements. We have found that individual 14-3-3ζ variants have different thermal stability that depends mostly on their oligomeric state. Phosphorylation of Ser58 destabilized the 14-3-3ζ protein significantly. The melting temperature (T_m_) of pζ decreased by approx. 10°C in comparison with ζ (Table 3; Figure 5A). The stability of monomeric ζm and phosphomimicking mutants ζ_S58D and ζ_S57D_S58D was also affected but the decline in T_m_ of 6–7°C was less prominent. The phosphomimicking mutation S58E lowered the T_m_ value of 14-3-3ζ by 2–3°C. Interestingly, the unfolding of pζ and the rather monomeric variants was more gradual and spread over a broader temperature range, compared to the dimeric variants (Figure 5A). For all studied variants, the heat denaturation was irreversible.

**TABLE 3:** Thermodynamic parameters of 14-3-3ζ variants. Melting temperatures and enthalpies were measured in triplicates. ΔH_vH_ is expressed per mole of the cooperative unit corresponding to the most populated oligomeric state of the 14-3-3ζ variant, i. e. dimer for ζ and ζ_S58E or monomer for ζ_S58D, ζ_S57D_S58D, ζm and pζ.

14-3-3	T_m_ (°C) nanoDSF	T_m_ (°C) DSC	ΔH_cal_ (kcal/mol)	ΔH_vH_ (kcal/mol)
ζ	60.35 ± 0.15	60.28 ± 0.01	105.33 ± 0.58	195.50 ± 0.87
ζ_S58E	58.19 ± 0.09	57.64 ± 0.01	118.33 ± 0.58	172.17 ± 0.29
ζ_S58D	54.21 ± 0.03	53.89 ± 0.01	106.00 ± 1.00	206.00 ± 1.00
ζ_S57D_S58D	53.58 ± 0.10	53.70 ± 0.01	101.33 ± 0.58	184.33 ± 0.58
ζm	53.04 ± 0.12	53.34 ± 0.02	80.47 ± 3.59	204.00 ± 5.00
pζ	50.70 ± 0.11	50.88 ± 0.02	74.83 ± 0.55	140.67 ± 0.58

DSC experiments enabled further determination of the thermodynamic parameters, such as unfolding enthalpies. All studied proteins displayed non-two-state transition behavior during denaturation and thus the calorimetric enthalpy (ΔH_cal_) and van’t Hoff enthalpy (ΔH_vH_) were obtained (Table 3). The calorimetric enthalpies of both pζ and ζm were lower than ΔH_cal_ of ζ and the phosphomimicking mutants. ΔH_vH_ of all proteins was larger than ΔH_cal_ suggesting cooperativity during protein unfolding.

### Diverse Effects of Selected Anions on Thermal Stability of 14-3-3ζ Variants

Protein stability may be affected by particular buffer composition. In this study, we investigated the impact of three sodium salts, commonly present in buffer formulations, on 14-3-3ζ protein thermal stabilities. 14-3-3ζ stability was assessed in the presence of chloride (Cl^−^), sulphate (SO_4_^2−^) and phosphate (HPO_4_^2−^) anions at four different concentrations. Based on good agreement between T_m_ values measured by DSC and nanoDSF (Table 3), we selected nanoDSF, as a high-throughput method, for this screening.

Sulphate and phosphate ions mostly exhibited a stabilizing effect with increasing concentration (Figure 5B). Stabilization as high as ΔT_m_ = 11.6°C was detected for ζ_S58E in 600 mM phosphate. A similar response was observed for the dimeric ζ (ΔT_m_ ∼ 9°C). On the contrary, for proteins with the dimer-monomer equilibrium shifted towards monomers, the stabilizing effect of certain anions was not that significant. In some cases, destabilization even occurred, i. e. for pζ, mζ and ζ_S57D_S58D (Figure 5B). A major difference in behavior was seen with the chloride ions. Whilst dimeric ζ and ζ_S58E were stabilized in all concentrations, a remarkable decrease in T_m_ was measured for monomerization-inducing constructs. The effect was strongest in case of pζ, which was destabilized by chloride in the whole concentration range, most substantially at the lowest (50 mM) chloride concentration (Figure 5B).

### Phosphorylation Significantly Increases 14-3-3ζ Hydrophobicity

The protein dimeric interface is composed mostly of residues forming salt bridges and conserved hydrophobic interactions. Based on the assumption that 14-3-3ζ monomerization should result in solvent exposure of hydrophobic residues (Sluchanko et al., 2011b), we investigated the hydrophobicity of all 14-3-3ζ variants. The fluorescent probe BisANS, sensitive to the polarity of its environment, was titrated to the protein and changes in BisANS fluorescence at 495 nm were measured, similarly to studies by Sluchanko and co-authors (Sluchanko et al., 2008; Sluchanko et al., 2011b). BisANS fluorescence intensity increased in the order ζ ≤ ζ_S58E << ζ_S57D_S58D < ζ_S58D < ζm << pζ (Figure 5C).

### Phosphorylation Modulates 14-3-3ζ Cellular Localization in HeLa and U251 Cells

Post-translational modifications have frequently been observed to change the attributes of proteins due to modified intracellular localization or trafficking (Michel et al., 1993; Humphries et al., 2008; Jin et al., 2014). We were interested to see if there is a functional impact of 14-3-3ζ Ser58 phosphorylation on protein expression *in vivo*. Therefore, we performed immunofluorescence colocalization experiments. Endogenous expression levels of ζ and pζ were detected in HeLa 1.3 cells and U251 cells, using specific anti-14-3-3ζ and anti-14-3-3ζ phospho-S58 primary antibodies.

**FIGURE 6:**
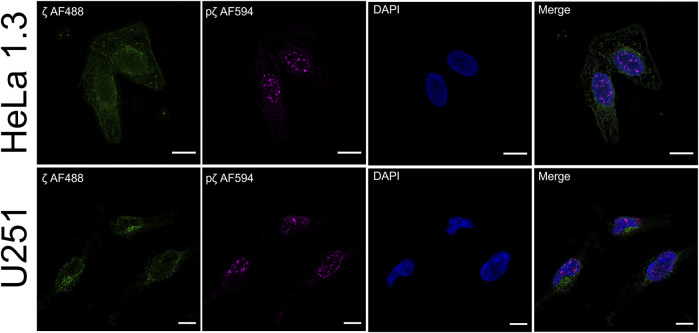
Phosphorylation of 14-3-3ζ shifts its localization from the cytosol to the nucleus. Endogenous expression levels of ζ (green) and pζ (magenta) were analyzed using immunofluorescence in HeLa 1.3 cells and U251 cells. Immunostaining was performed using specific primary anti-14-3-3ζ and anti-14-3-3ζ pS58 antibodies, and corresponding AF-conjugated secondary antibodies. Nuclei were stained with DAPI (blue). Scale bars represent 10 µm. Negative controls were performed to exclude nonspecific binding of primary antibodies (Supplementary Figure S6).

Using our setup, we were able to observe different expression patterns with respect to 14-3-3ζ phosphorylation (Figure 6). We noticed a rather diffuse presence of ζ in the cytoplasm with non-specific occurrence of foci in both cell lines. There was no notable expression seen in the cell nucleus. Surprisingly, the expression of pζ was recorded almost exclusively in the nucleus of both cell lines as distinct strong foci, with minimal expression in the cytoplasm (Figure 6). We did not detect any significant overlap between the fluorescence signals for ζ and pζ.

## Discussion

### Preparation of 14-3-3ζ Phosphorylated at Ser58

The phosphorylation of Ser58 and its effects on protein properties and oligomeric state has been an important unresolved issue in the community. The ambiguity of reported observations, especially their impact on the oligomeric state (Powell et al., 2002; Woodcock et al., 2003; Gu et al., 2006), motivated us to find a way to prepare 14-3-3ζ protein phosphorylated at Ser58 in high purity and yield for its biophysical analysis. In past studies, 14-3-3ζ protein could not be completely phosphorylated under the presented experimental conditions (Powell et al., 2003; Shen et al., 2003; Sluchanko and Uversky, 2015; Woodcock et al., 2018). Here, we have established a protocol for the preparation of 14-3-3ζ protein specifically and fully phosphorylated at Ser58.

Phosphorylated 14-3-3ζ protein was prepared by co-expression of 14-3-3ζ and PKA in *E. coli*. This technique has been successfully employed in other studies for *in vivo* phosphorylation of proteins in prokaryotic cells (Sluchanko et al., 2017; Tugaeva et al., 2017). Despite optimization of conditions, phosphorylation of 14-3-3ζ at Ser58 has not reached 100% in our hands, as reported elsewhere (Powell et al., 2003; Shen et al., 2003; Sluchanko and Uversky, 2015; Woodcock et al., 2018), likely due to limited accessibility of Ser58 to PKA and/or the activity of *E. coli* phosphatases (Kennelly, 2002). 14-3-3ζ phosphorylated at Ser58 was separated from the non-phosphorylated fraction by AEX chromatography. pζ was eluted first (Figure 1B), since at these experimental conditions pζ exists as a monomer with approx. overall charge −17e, whereas ζ is a dimer with overall charge −30e (−15e/monomer).

Besides full phosphorylation at Ser58, minor phosphorylation (∼4%) was detected at position Ser28, although the sequence ‘AMKpS^28^V’ does not correspond to an ideal PKA recognition motif. It should also be mentioned that our 14-3-3ζ constructs contain an artificial C25A mutation in this short region, to avoid the formation of intermolecular disulphide bridges. This mutation could possibly influence PKA specificity.

The co-expression approach enabled the preparation of pure pζ in milligram quantities. To obtain similar yields *in vitro* (Gu et al., 2006; Powell et al., 2003; Woodcock et al., 2003, 2010), this would require a prior purification of the 14-3-3ζ protein in high amounts and the purification or purchase of protein kinase at substantial cost. Moreover, the protein must be subjected to the phosphorylation reaction and additional purification steps must be performed to recover pζ. Besides, problems with aggregation of the *in vitro* prepared pζ have been reported (Sluchanko and Uversky, 2015).

The presented *in vivo* phosphorylation assay is a more convenient alternative to *in vitro* assays since it increases time efficiency and yield. Protein expression and phosphorylation proceed in the cell simultaneously, and the phosphorylated protein can be isolated directly from the soluble fraction of the cell lysate. In addition, an analogous protocol may be used to phosphorylate other 14-3-3 isoforms (β, γ, ε, η), while only minor optimization of expression conditions should be necessary.

### Phosphorylation Has a Different Impact on 14-3-3ζ Oligomeric State Than Phosphomimicking Mutations

We have demonstrated that the studied 14-3-3ζ variants prefer distinct oligomeric states at physiologically relevant concentrations (Figure 3). In addition, we have determined their dimerization dissociation constants (Table 2). The mutually consistent qualitative and quantitative results were obtained by multiple biophysical methods such as AUC, SEC-RALS, native-PAGE and FRET (Figure 3, Figure 4). The provided K_D_ values allow one to calculate the monomeric (M) and dimeric (D) fractions of 14-3-3ζ variants at any concentration using the derived Equation 4. For illustration, the dimer-monomer populations of 14-3-3ζ proteins at the estimated physiological concentration (25 μM) are listed in the last two columns of Table 2.

We would like to note that for those 14-3-3ζ variants, whose determined K_D_ values were out of the concentration range used in SEC-RALS experiments (7.3–183 μM), i. e. for pζ and ζm (K_D_ in mM range), we observed an artificial “concentration dependence of extracted K_D_ values” (data not shown). Therefore, we calculated these K_D_ values from the dimer-monomer populations at the highest measured concentration (183 μM), where the expected errors are the lowest. Still, the listed values in Table 2 should be considered as estimates rather than accurate values.

Despite the mentioned shortcoming, the dimerization K_D_ values for individual 14-3-3ζ variants differ by several orders of magnitude (Table 2). We have shown that the K_D_ for pζ is ∼8 mM in contrast to the phosphomimicking mutants used in the past, i. e. ζ_S58E (Gu et al., 2006; Sluchanko et al., 2008; Sluchanko et al., 2011a) and ζ_S58D (Powell et al., 2003; Haladová et al., 2012; Civiero et al., 2017; Woodcock et al., 2018), whose K_D_ values we determined to be ∼0.35 and 130 μM, respectively. The monomerization of ζ_S58E at very low micromolar concentration has been reported by Sluchanko and co-workers (Sluchanko et al., 2008; Sluchanko et al., 2011b). On the contrary, Gu et al. (2006) did not observe the formation of any ζ_S58E dimers. To compare the commonly used mutants from past works, the lower monomerization potency of S58E mutation in comparison to S58D is likely caused by the longer and more flexible side chain of Glu, which allows for an easier formation of 14-3-3 dimers.

The significant difference in the dimerization K_D_ values between pζ and the phosphomimicking mutants calls into question the outcomes of studies where such phosphomimicking mutations were used. Particularly, results obtained with the ζ_S58E mutant should be taken with a dose of caution (Gu et al., 2006; Sluchanko et al., 2008; Sluchanko et al., 2011a).

Although we have described an approach for preparation of pζ in high purity and yields, we can imagine experimental setups when the mutated variants may be preferred. In such cases, we recommend using the double phosphomimicking mutant ζ_S57D_S58D with a K_D_ ∼350 μM, which possesses the closest dimerization K_D_ value of any listed phosphomimicking mutant to the truly phosphorylated variant. In other cases, when the negative charge at the dimeric interface is not essential and a pζ-like monomeric preference is rather desired, we recommend employing the monomeric mutant ζm, with the K_D_ of ζm being ∼5 mM comparable to pζ.

Despite the varying oligomeric preferences among the studied 14-3-3ζ variants, their secondary structure as inspected by CD spectroscopy is very similar. Overall predicted α-helical content (79–85%) was in accordance with helicity determined from known crystal structures of 14-3-3ζ protein (Gardino et al., 2006). MRE at 222 nm for all proteins ranged between −23,000 and −27,000 deg cm^2^ dmol^−1^ (Figure 2B), which agrees with previously published data for 14-3-3ζ protein (Sluchanko et al., 2008; Sluchanko and Uversky, 2015; Woodcock et al., 2018).

A decrease in MRE by ∼10% observed for pζ may indicate minor changes in the pζ structure. Sluchanko and Uversky (2015) and Woodcock et al. (2018) proposed the unfolding of ∼40 residues in the N-terminal helices. On the contrary, we did not observe any changes in MRE magnitude for ζ_S58E and ζ_S58D, as previously reported to be decreased by ∼12–16 and ∼12%, respectively (Sluchanko and Uversky, 2015; Woodcock et al., 2018). We hypothesize that discrepancies in these data may be caused by problems with measurement of accurate protein concentration due to different modifications at Ser58 (phosphorylation or mutations). Ser58 is in juxtaposition to Trp59, one of two tryptophanes that are the main contributors to the overall absorbance at 280 nm, which is used for the protein concentration determination.

### Distinct Dimer-Monomer Equilibria Affect the Thermostability and Hydrophobicity of 14-3-3ζ Variants

Differences in the oligomeric state of 14-3-3ζ variants also impacted their thermal stability. Measured melting temperatures demonstrated that monomerization on its own significantly contributes to protein destabilization, but phosphorylation decreases the thermal stability to an even greater extent (Table 3). This was also observed after phosphorylation of the monomeric mutant ζm, whose T_m_ decreased to comparable level as for pζ (data not shown). This suggests that phosphorylation might, in addition to monomerization, cause additional changes in the protein structure and stability.

The melting temperature of ζ (∼60°C) and the phosphomimicking mutants ζ_S58E (∼58°C) and ζ_S58D (∼54°C) agreed well with published studies (Sluchanko et al., 2008, 2012; Woodcock et al., 2018). A mild thermal destabilization observed for ζ_S58E correlates with the preferred dimeric arrangement. Interestingly, melting temperatures of monomeric mutants examined here or reported in the literature were always fluctuating around 53 °C (Sluchanko et al., 2011b; Sluchanko and Uversky, 2015), but none of the mutations destabilized 14-3-3ζ to the same extent as phosphorylation (Table 3).

From the DSC measurements, we also obtained enthalpies accompanying the protein unfolding (Table 3). Van’t Hoff enthalpy higher than calorimetric enthalpy indicates that unfolding of 14-3-3ζ proteins is a non-two-state process, which is accompanied by cooperation. We note, however, that all studied variants exhibited irreversible denaturation, and therefore one cannot confidently use the analysis that is valid for reversible thermodynamics.

Next, we inspected the effect of common salts on thermal stability of 14-3-3ζ variants. In general, sulphate and phosphate were shown to stabilize 14-3-3ζ proteins, while the impact of chloride was much smaller or even destabilizing (Figure 5B). This is in good agreement with the Hofmeister series (Hofmeister, 1888; Kunz et al., 2004) and observations for 14-3-3γ (Bustad et al., 2012).

In case of the preferentially monomeric proteins, and especially pζ, we observed destabilization of the protein in the presence of sodium chloride (Figure 5B). We hypothesize that chloride may interact unfavorably with the exposed hydrophobic patches, as its interaction with water is substantially weaker than both phosphate and sulphate. Even though the sulphate anion is considered more stabilizing than phosphate according to the Hofmeister series (Hofmeister, 1888), 14-3-3ζ proteins exhibited greater stabilization by the latter (Figure 5B). We speculate, that possible binding of the phosphate ion into the phospho-peptide binding groove of 14-3-3 would increase its stability, which is in agreement with the overall phospho-target binding nature of the 14-3-3 family (Yaffe et al., 1997; Obsil and Obsilova, 2011).

**FIGURE 7:**
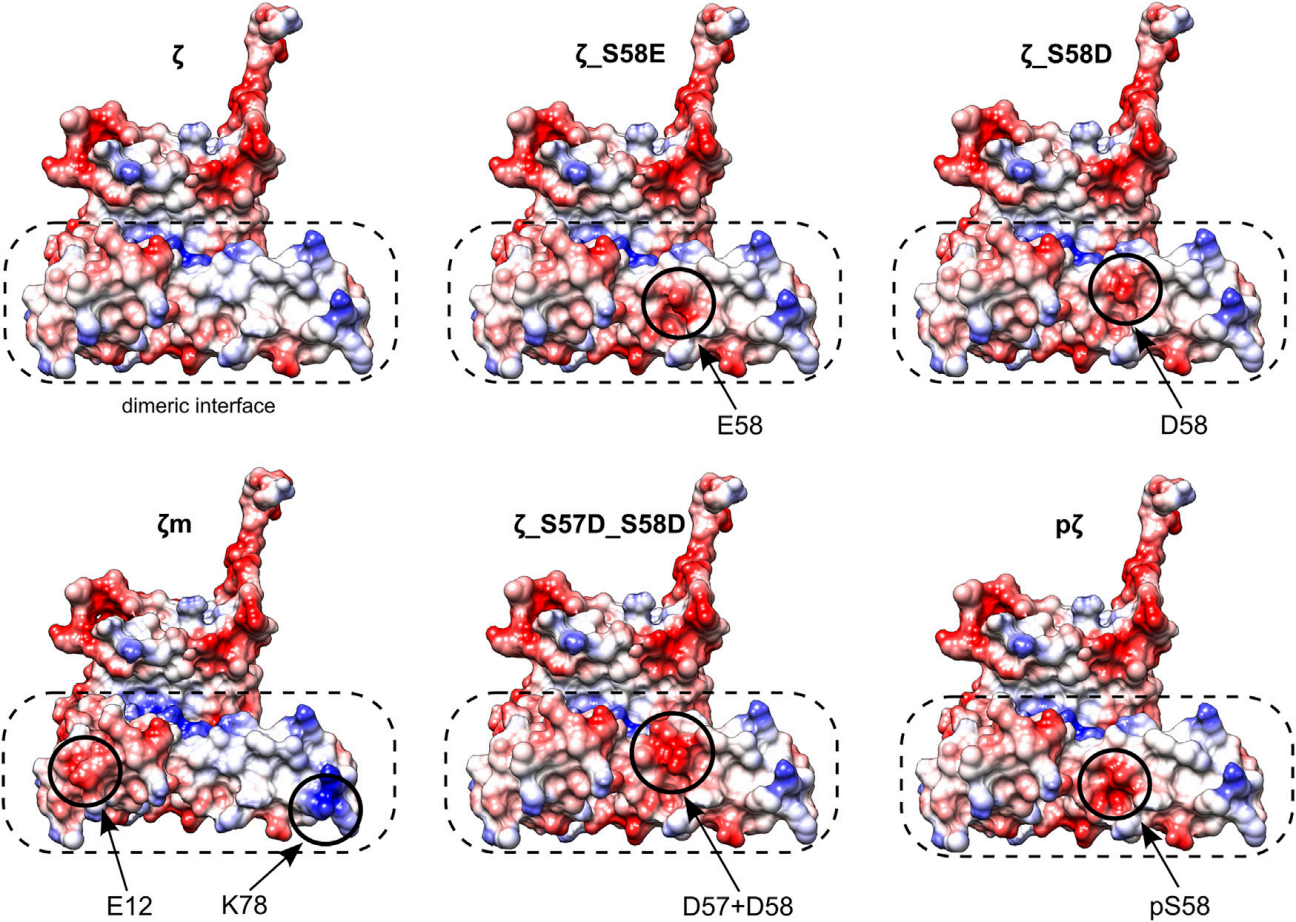
Electrostatic potential maps of all 14-3-3ζ variants. Only monomeric subunits are shown. Modifications and corresponding changes in charge distributions at the dimeric interface (in a dashed rectangle) are highlighted (in solid circles). Negative electrostatic potential is depicted in red, positive in blue. Atom coordinates of the 14-3-3ζ were adopted from Kostelecky et al. (2009) and Nagy et al. (2017). The desired residues were mutated *in silico* and their conformations were selected from a library of possible conformers. Mutagenesis and generation of the electrostatic potential maps with Coulombic surface coloring were performed in UCSF Chimera (Pettersen et al., 2004).

Different monomerization tendencies among 14-3-3ζ variants were reflected in protein hydrophobicity, as well. In general, the preferentially monomeric 14-3-3ζ variants exhibited higher affinity to the fluorescent probe BisANS than dimers. We attribute the distinct affinity towards BisANS to two main factors. First, monomerization results in the exposure of hydrophobic patches that were originally hidden at the dimeric interface. Therefore, monomeric variants appear to be more hydrophobic, similarly to previous studies (Sluchanko et al., 2008, 2014; Sluchanko et al., 2011b; Woodcock et al., 2018). Second, the affinity to BisANS is influenced by differences in the charge distribution at the dimeric interface between variants, as demonstrated on the electrostatic potential maps (Figure 7).

Considering those two factors, the overall trends seen in Figure 5C appear to be in line with the dimerization K_D_ values, with two outstanding distinctions. First, the affinity towards BisANS is higher for ζ_S58D compared to ζ_S57D_S58D, although the double phosphomimicking mutant is slightly more monomeric (Table 2). This behavior may be explained by the higher negative charge of ζ_S57D_S58D (Figure 7) and thus larger repulsion of the BisANS probe that is also partially negatively charged. Second, the hydrophobicity of pζ seems much higher than of ζm, despite similar dimerization K_D_ values (Table 2). During the design of the monomeric mutant ζm, we replaced two hydrophobic residues by charged amino acids (namely L12E_M78K) (Jandova et al., 2018), which reduced the size of hydrophobic patches (Figure 7). On the contrary, these hydrophobic patches are preserved within pζ, making pζ more hydrophobic.

### Phosphorylation of 14-3-3ζ Alters its Subcellular Localization in HeLa 1.3 and U251 Cells

Immunofluorescence experiments revealed a clear difference in expression levels of ζ and pζ with respect to subcellular compartments in the tested cell lines. ζ was mostly found dispersed throughout the cytoplasm, while pζ was seen in the nucleus in the form of strong foci (Figure 6). Our results suggest that upon phosphorylation, pζ is transported and concentrated in the nucleus by an unknown mechanism. Hence, we deduce that pζ has a differing role to ζ in the cell, perhaps in regulation of the cell cycle or genome transcription, as reported elsewhere (Hermeking and Benzinger, 2006; Hou et al., 2010; Tzivion et al., 2011). A connection to changes in the oligomeric state of the protein may not be excluded. Note that the cell lines used in this study are derived from cervical carcinoma (HeLa 1.3) (Takai et al., 2010) and glioblastoma tumor cells (U251) (Westermark et al., 1973), thus the described localization pattern might be specific to cancer cells. The presented results will be corroborated in future work by testing additional cell lines, and by pull-down experiments coupled with mass spectrometry analysis, to address the varying functional roles of ζ and pζ by looking at binding partners. Immunofluorescence experiments with cells transiently transfected with ζ_S57D_S58D and ζm will likely shed light on the question whether the nuclear translocation is driven primarily by the additional negative charge or monomerization.

We did not notice double labelling of the same spots during immunofluorescence, which could be expected depending on the specificity of the used antibodies. To this end, we tested their specificity by Western blotting (Supplementary Figure S7). When purified proteins were blotted, both ζ and pζ were detected by the anti-14-3-3ζ antibody, but only pζ was detected by the anti-14-3-3ζ pS58 antibody, confirming its specificity (Supplementary Figure S7A). When we blotted cell lysates with the same antibodies, we did not record any corresponding signal with the anti-14-3-3ζ pS58 antibody, but clear bands were seen when using the anti-14-3-3ζ antibody (Supplementary Figure S7B). Even though pζ could not be detected in the whole cell lysates by Western blotting it could be visualized by immunofluorescence as distinct strong foci using the anti-14-3-3ζ pS58 antibody. However, using the anti-14-3-3ζ antibody which could visualize both variants (Supplementary Figure S7A), we could detect only a diffuse signal that prevailed in the cytoplasm and no distinct foci in the nucleus (Figure 6). The absence of pζ foci when using the anti-14-3-3ζ antibody can be explained by high levels of ζ with respect to pζ which could compromise detection of the pζ variant.

## Conclusion

Our case study, focused on 14-3-3ζ protein, demonstrates that phosphomimicking mutants are quite poor substitutes for the phosphorylated variant. Two phosphomimicking mutants used in previous studies, namely ζ_S58E and ζ_S58D, and a novel double mutant ζ_S57D_S58D were compared with 14-3-3 pζ. Significant differences were revealed in the dimerization dissociation constants between the phosphomimicking mutants ζ_S58E, ζ_S58D, and ζ_S57D_S58D (∼0.35, 132, 348 μM) and the phosphorylated variant (∼7.6 mM). The dissimilarity in the oligomeric states was also reflected in the melting temperatures of the phosphomimicking mutants (T_m_ ∼ 57.6, 53.9, 53.7°C) with respect to the phosphorylated variant (T_m_ ∼ 50.9°C). Ser58 phosphorylation also increased the hydrophobicity more than two times compared to the phosphomimicking mutants. Moreover, phosphorylation changed the cellular localization of 14-3-3ζ from the cytoplasm towards the nucleus. For future studies of 14-3-3 phosphorylation, when the use of phosphorylated protein is not applicable, e. g. *in vivo* experiments, we recommend utilizing the double mutant ζ_S57D_S58D, as its behavior was most similar to pζ. When the adequate oligomeric state is more important than the negative charge at the dimeric interface, we would opt for the monomeric mutant ζm. In conclusion, our findings highlight the importance of careful mutant design and encourage the verification of their properties with the original modification.

## Data Availability

The datasets presented in this study can be found in the Figshare repository (https://doi.org/10.6084/m9.figshare.c.5813213.v1).

## Footnotes

1
https://bitc.sr.unh.edu

2
https://pax-db.org/
