# Supplementary material for: Phosphorylated and Phosphomimicking Variants May Differ—A Case Study of 14-3-3 Protein
Source: Front Chem. 2022 Mar 7;10:835733. doi: 10.3389/fchem.2022.835733 (PMC8935074; doi:10.3389/fchem.2022.835733)
Supplement: Supplementary file 1 [file DataSheet1.pdf]

Supplementary Material

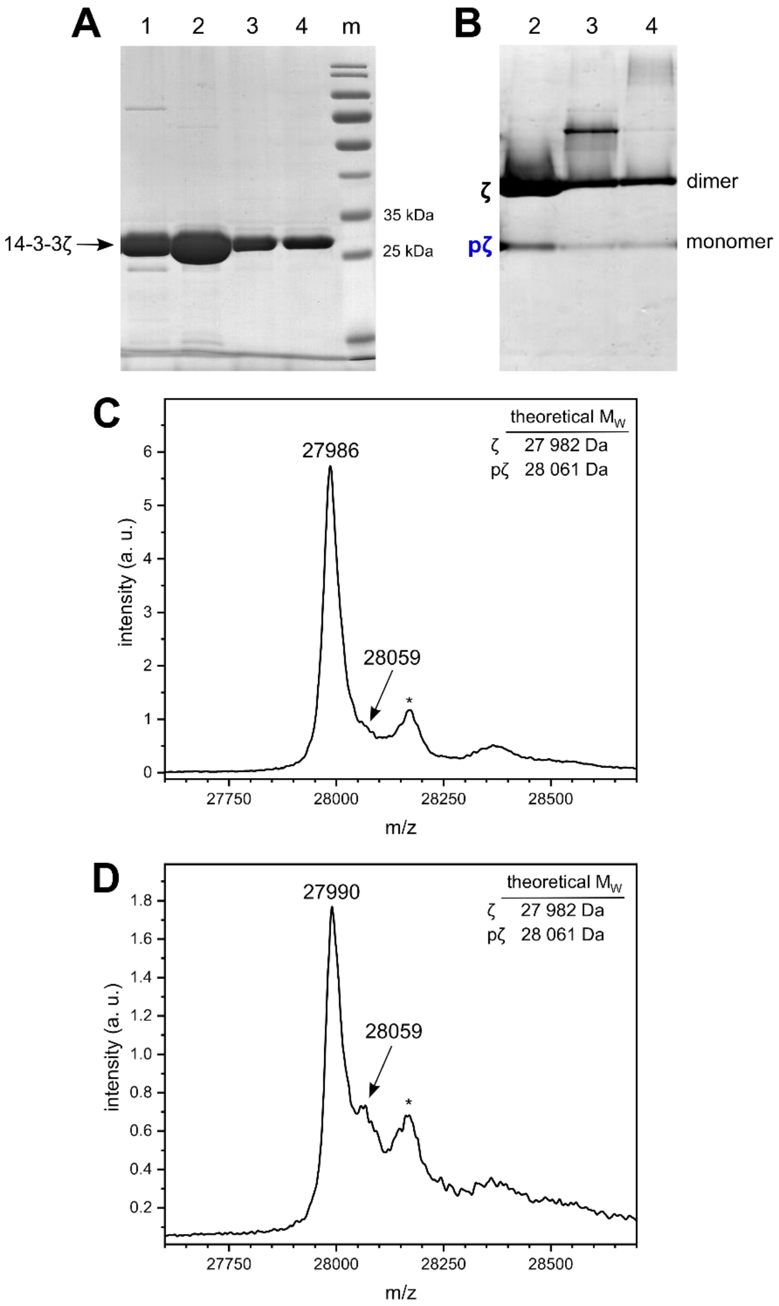

Supplementary Figure S1: Characterization of eluted fractions from AEX. (A) SDS-PAGE analysis of fractions eluted at 1) 25 mS/cm, 2) 28–30 mS/cm, 3) 34 mS/cm, 4) 37 mS/cm. **(B)** Native-PAGE analysis of fractions 2)–4). **(C)** MALDI-TOF MS spectrum of the fraction 2) (i. e. ζ) showing minor contamination by pζ. **(D)** MALDI-TOF MS spectrum of the fractions 3+4) containing a mixture of ζ and pζ. Theoretical M_W(ζ) = 27 982 Da, theoretical M_W(pζ) = 28 061 Da. Asterisk denotes a protein adduct with the MALDI matrix (ferulic acid, +176).

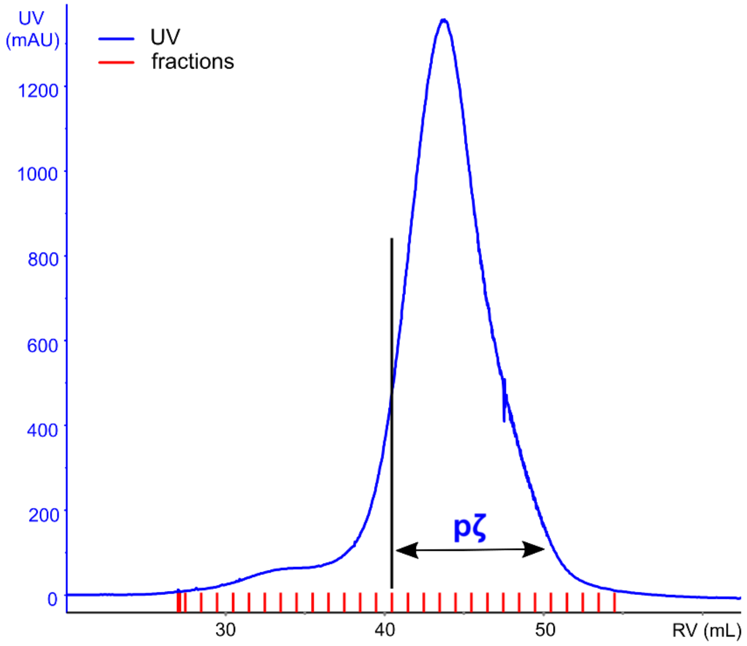

Supplementary Figure S2: Chromatogram of the SEC step. Fractions corresponding to the final pζ sample are depicted.

Results of the LC-MS/MS analysis of 14-3-3ζ phosphorylated at Ser58

The LC-MS/MS analysis covered 97% of the protein sequence with two serines (Ser28 and Ser58) being found to be phosphorylated. All signals (41 of 41 recorded tandem mass spectra) assigned to peptides containing Ser58 indicated quantitative phosphorylation of this site. Only 4% of signals of peptides containing Ser28 (5 of 116 recorded tandem mass spectra) were found to correspond to their phosphorylated forms, while the remaining signals were assigned to non-phosphorylated peptides.

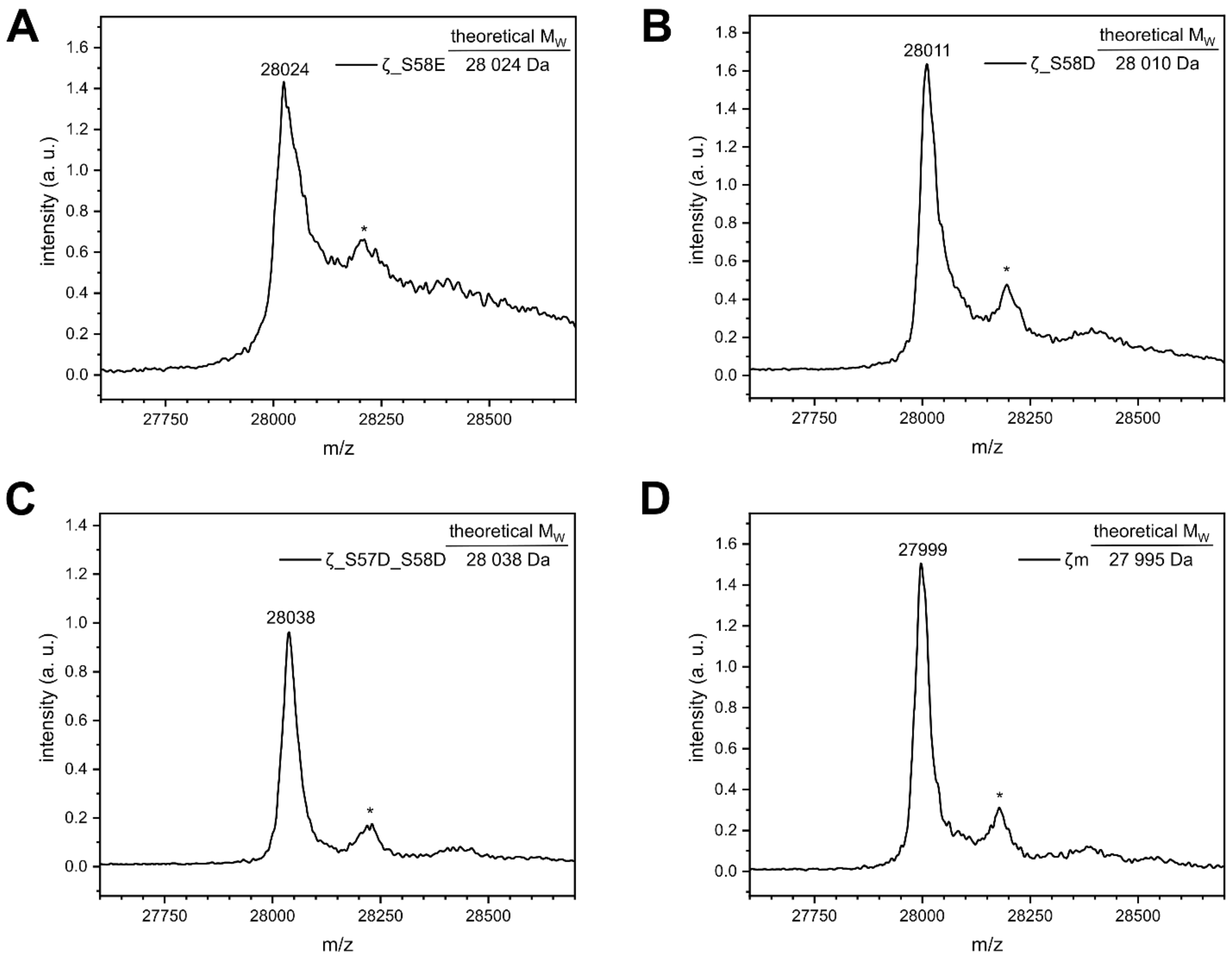

Supplementary Figure S3: MALDI-TOF MS spectra of purified 14-3-3ζ variants. (A) ζ_S58E, **(B)** ζ_S58D, **(C)** ζ_S57D_S58D, **(D)** ζm. Asterisk denotes a protein adduct with the MALDI matrix (ferulic acid, +176).

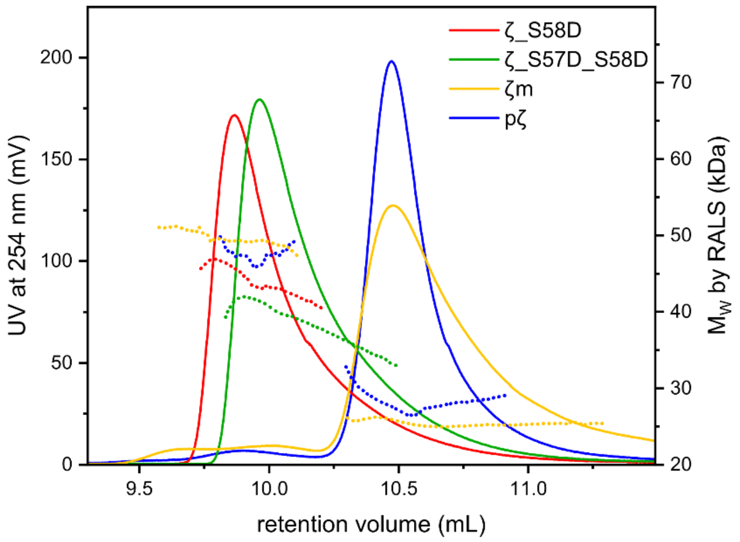

Supplementary Figure S4: SEC-RALS data for determination of the dimerization dissociation constants of 14-3-3ζ variants. The SEC-RALS experiment was performed for 164 µM ζ_S58D and ζ_S57D_S58D and 183 µM ζm and pζ at 20°C.

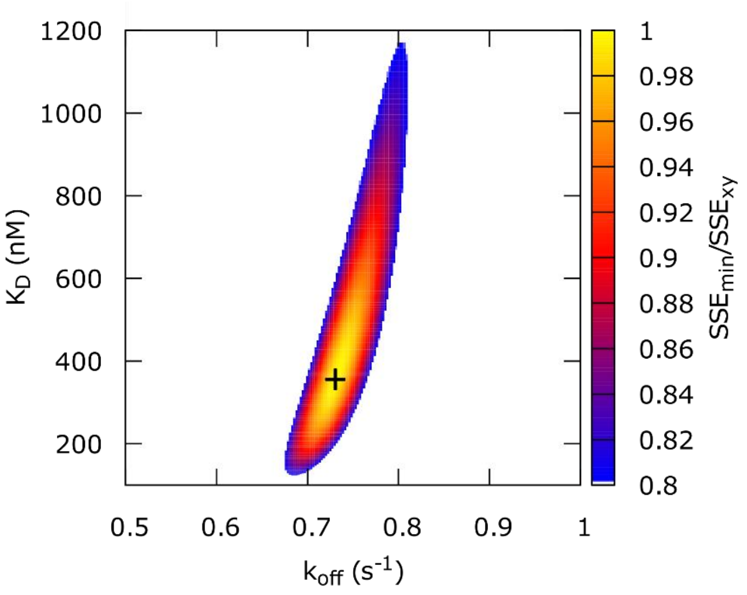

Supplementary Figure S5: Heatmap analysis of the FRET experiment used for determination of the ζ_S58E K_D. Dependence of sum square error (SSE) on the dissociation rate constant k_{off} and K_D from the FRET assay for ζ_S58E variant. Minimum SSE (indicated by '+') divided by the SSE obtained at each x, y coordinate (SSE_{min}/SSE_{xy}), i. e. for each combination of k_{off} and K_D, is shown.

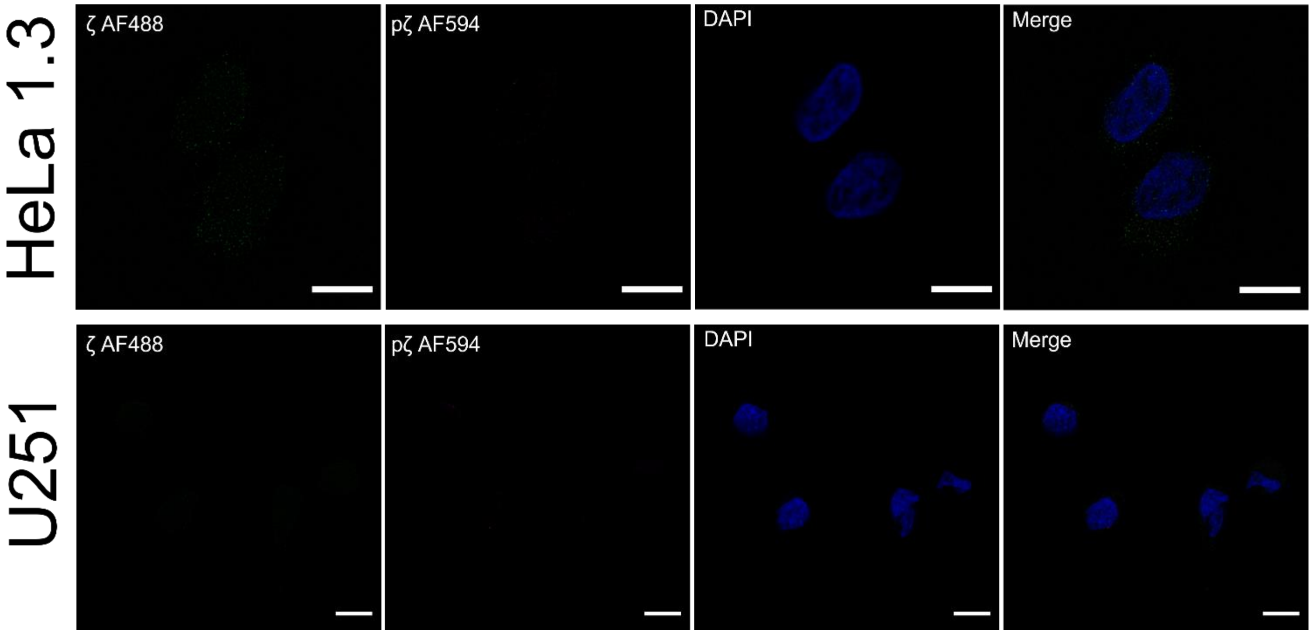

Supplementary Figure S6: Immunofluorescence of negative controls. Immunostaining was performed using AF-conjugated secondary antibodies without using the primary antibodies to exclude nonspecific binding. Nuclei were stained with DAPI (blue). Scale bars represent 10 µm.

Western Blotting procedure

HeLa 1.3 and U251 cells were harvested from T-25 flasks and washed twice with PBS. Cell pellets were lysed in RIPA buffer (50 mM Tris-HCl, pH 7.4, 150 mM NaCl, 1% Triton X-100, 0.5% sodium deoxycholate, 0.1% SDS, 1 mM EDTA, 10 mM NaF and Protease Inhibitor Cocktail (cOmplete, Roche)) for 45 minutes at 4°C. Samples were then mixed with 4x SDS-loading dye and applied onto an 10% SDS-PAGE gel. After transferring the resolved proteins to a nitrocellulose membrane (Amersham Protran 0.45 µm NC, GE Healthcare) by wet transfer, the membrane was blocked in 3.5% non-fat milk diluted in TBS supplemented with 0.2% Tween 20 (TBS-T) for 1 hour at room temperature.

After washing 3 times with TBS-T, the membrane was incubated with a primary antibody diluted in 3.5% milk in TBS-T 1:2000 (Anti-14-3-3ζ (phospho S58) antibody (ab51109, Abcam) or 14-3-3ζ Antibody (MA5-37641, Invitrogen)) for 1 h at room temperature. After washing 3 times with TBS-T, the membrane was incubated with a secondary antibody diluted in 3.5% milk in TBS-T 1:5000 (Goat Anti-Rabbit IgG H&L, ab97051 or Goat Anti-Mouse IgG (Fc specific)-Peroxidase antibody, A0168) for 1 h at room temperature. After washing 3 times with TBS-T buffer, proteins were detected using the SuperSignal West Femto Maximum Sensitivity Substrate system (ThermoFisher Scientific).

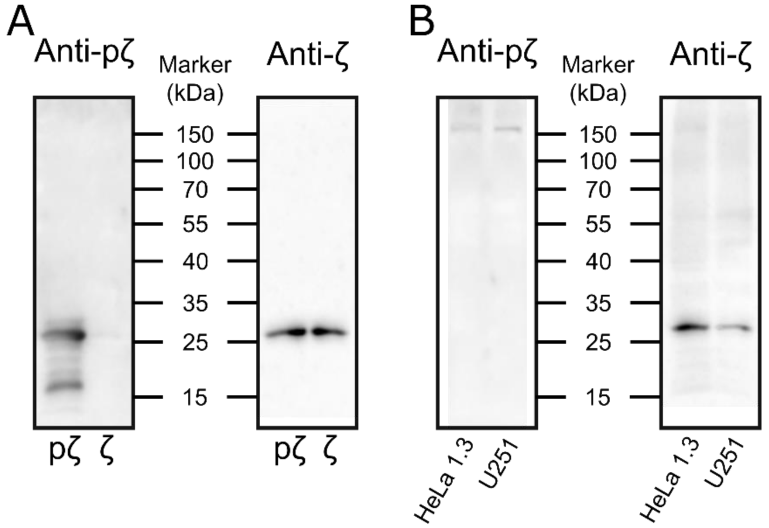

Supplementary Figure S7: Western blot analysis of recombinant ζ and pζ proteins and cell lysates. (A) Purified proteins were detected with both used primary antibodies and a corresponding secondary antibody. **(B)** Lysates of HeLa 1.3 and U251 cells were detected with both used primary antibodies and a corresponding secondary antibody.

Supplementary Table S1: Melting temperatures of 14-3-3ζ variants in 20 mM HEPES pH 8 without presence of salt.

14-3-3	T_m (°C)
**ζ**	59.99 ± 0.01
**ζ_S58E**	56.32 ± 0.05
**ζ_S58D**	53.30 ± 0.03
**ζ_S57D_S58D**	53.30 ± 0.03
**ζm**	53.59 ± 0.06
**pζ**	52.22 ± 0.10
